# DNA barcoding of *Corydalis*, the most taxonomically complicated genus of Papaveraceae

**DOI:** 10.1002/ece3.4886

**Published:** 2019-01-21

**Authors:** Feng‐Ming Ren, Ying‐Wei Wang, Zhi‐Chao Xu, Ying Li, Tian‐Yi Xin, Jian‐Guo Zhou, Yao‐Dong Qi, Xue‐Ping Wei, Hui Yao, Jing‐Yuan Song

**Affiliations:** ^1^ Key Lab of Chinese Medicine Resources Conservation, State Administration of Traditional Chinese Medicine of the People's Republic of China, Institute of Medicinal Plant Development Chinese Academy of Medical Sciences, Peking Union Medical College Beijing China; ^2^ Chongqing Institute of Medicinal Plant Cultivation, Research and Utilization on Characteristic Biological Resources of Sichuan and Chongqing Co‐construction Lab Chinese Medicine Breeding and Evaluation Engineering Technology Research Center of Chongqing Chongqing China; ^3^ Beijing Botanical Garden, Institute of Botany Chinese Academy of Sciences Beijing China

**Keywords:** biodiversity hotspot, *Corydalis*, DNA barcoding, *matK*, species identification, the internal transcribed spacer

## Abstract

The genus *Corydalis* is recognized as one of the most taxonomically challenging plant taxa. It is mainly distributed in the Himalaya–Hengduan Mountains, a global biodiversity hotspot. To date, no effective solution for species discrimination and taxonomic assignment in *Corydalis* has been developed. In this study, five nuclear and chloroplast DNA regions, ITS, ITS2, *matK*, *rbcL*, and *psbA‐trnH*, were preliminarily assessed based on their ability to discriminate *Corydalis* to eliminate inefficient regions, and the three regions showing good performance (ITS, ITS2 and *matK*) were then evaluated in 131 samples representing 28 species of 11 sections of four subgenera in *Corydalis* using three analytical methods (NJ, ML, MP tree; K2P‐distance and BLAST). The results showed that the various approaches exhibit different species identification power and that BLAST shows the best performance among the tested approaches. A comparison of different barcodes indicated that among the single barcodes, ITS (65.2%) exhibited the highest identification success rate and that the combination of ITS + matK (69.6%) provided the highest species resolution among all single barcodes and their combinations. Three Pharmacopoeia‐recorded medicinal plants and their materia medica were identified successfully based on the ITS and ITS2 regions. In the phylogenetic analysis, the sections *Thalictrifoliae*, *Sophorocapnos*, *Racemosae*, *Aulacostigma*, and *Corydalis* formed well‐supported separate lineages. We thus hypothesize that the five sections should be classified as an independent subgenus and that the genus should be divided into three subgenera. In this study, DNA barcoding provided relatively high species discrimination power, indicating that it can be used for species discrimination in this taxonomically complicated genus and as a potential tool for the authentication of materia medica belonging to *Corydalis*.

## 1 INTRODUCTION

**Figure 1:**
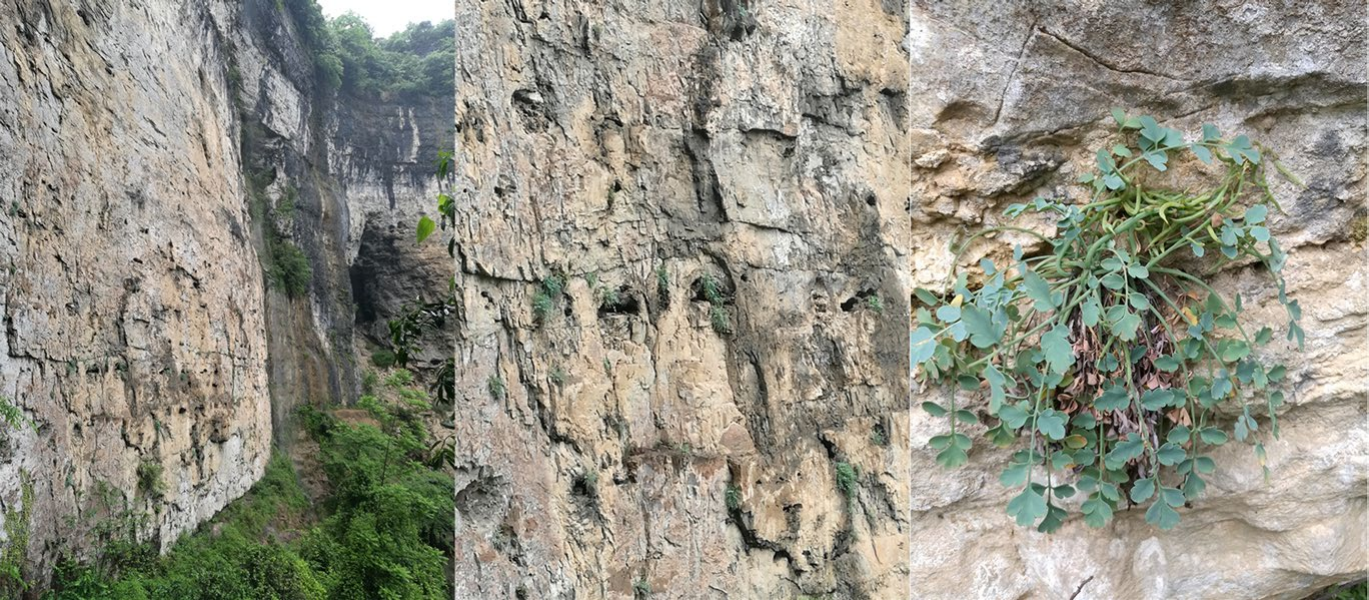
The specialized habitats of *Corydalis saxicola* in dry limestone cliffs

The Himalaya–Hengduan Mountains represent a global biodiversity hotspot with high levels of biodiversity and endemism and has recently become a priority conservation area due to the negative effects of climate change and intensive human activities in this region (Yan et al., 2014). *Corydalis DC*., the largest genus of Papaveraceae (Zhang, Su, & Liden, 2008), is an important component of the biodiversity in the Himalaya–Hengduan Mountains. This genus originated from the Hengduan Mountains and was recently distributed from this region to the Qinghai–Tibet Plateau (Linden, Fukuhara, & Axberg, 1995; Wu, 1996). Due to the complicated geological history of this region and the dramatic variations in its local climates and topography (Yan et al., 2014), as well as the reticulate evolution and intensive differentiation in the phylogenesis of *Corydalis* (Wang, 2006; Wu, 1996), this genus exhibits high levels of morphological and habitat diversity. Some species grow in specialized habitats, such as dry limestone cliffs (Figure 1) and alpine hillsides (Zhang et al., 2008), which are inaccessible. Most species exhibit complicated morphological characteristics. The leaves, subterranean organs, fruits, seeds, and particularly the floral structures of *Corydalis* species are very complex and show high variability, which seriously hampers accurate species discrimination and taxonomic assignment. Species identification is a precondition of biodiversity conservation and is also fundamental to almost all disciplines of botany (Chen et al., 2016). However, due to its complicated morphological characteristics, arduous procedures for sample collection, the absence of seasoned specialists, and the limitations of traditional morphology‐based taxonomy, *Corydalis *is recognized as one of the most taxonomically complicated plant taxa.

DNA barcoding, which uses one or several short standardized DNA regions for species identification (CBOL Plant Working Group, 2009; Hebert, Cywinska, Ball, & deWaard, 2003; Kress, Wurdack, Zimmer, Weigt, & Janzen, 2005), exhibits the outstanding advantage of not being affected by morphological characteristics. This technique has been widely applied for species identification (Barco, Raupach, Laakmann, Neumann, & Knebelsberger, 2016; Liu, Provan, Gao, & Li, 2012; Yan et al., 2014), the discovery of new or cryptic species (Huemer, Karsholt, & Mutanen, 2014; Liu, Möller, Gao, Zhang, & Li, 2011; Zemlak, Ward, Connell, Holmes, & Hebert, 2009), the assessment of biodiversity (Ji et al., 2013; Lahaye et al., 2008; Taberlet, Coissac, Pompanon, Brochmann, & Willerslev, 2012), the characterization of genetic diversity (Sucher & Carles, 2008; Zhang, Niu, Guo, Wang, & Eaton, 2015), and the identification of species used in traditional Chinese medicine (Chen et al., 2010; Yuan et al., 2015). DNA barcoding has been shown to be an effective method for a variety of applications that involve species identification (Yan et al., 2014), but its performance in this taxonomically complicated genus has not been evaluated.

Various regions in the nuclear and chloroplast genomes have been proposed as DNA barcodes for plants. The *psbA‐trnH* intergenic spacer region has been proposed as a DNA barcode for land plants (Kress et al., 2005). Portions of the plastid coding genes *rbcL* and *matK *have been suggested as the core barcodes to establish a barcoding database for plant species (CBOL Plant Working Group, 2009). The nuclear internal transcribed spacer (ITS), which has a high rate of nucleotide substitution and thus relatively high discrimination power, has been proposed to be incorporated into a core barcode for seed plants (China Plant BOL Group, 2011). The ITS2 region, a subregion of ITS, has been selected as a valuable sequence tag for the identification of medicinal plants and materia medica (Chen et al., 2010; Han et al., 2013; Yao et al., 2010). However, no single barcode for plants can perform as well as COI does in animals (Hollingsworth, Graham, & Little, 2011). In fact, individual barcodes usually exhibit unequal species discriminatory ability in different plant groups, and therefore, it is necessary to select appropriate barcodes for *Corydalis*. Furthermore, several different analytical methods, such as tree‐based, distance‐based, sequence similarity‐based, and character‐based methods, have been used for the assessment of species discrimination ability (Austerlitz et al., 2009; Frezal & Leblois, 2008; Li et al., 2011; Sandionigi et al., 2012; Yan et al., 2014). Different analytical methods typically show dissimilar species discrimination power on the same datasets (Kool et al., 2012; Li et al., 2011; van Velzen, Weitschek, Felici, & Bakker, 2012; Yan et al., 2014), but the discrimination ability of different analytical methods in *Corydalis* remains unknown.

An ideal DNA barcode should have a highly universal single primer pair, provide high‐quality bidirectional sequences, have a high discriminatory power among species (CBOL Plant Working Group, 2009; Kress et al., 2005; Lahaye et al., 2008), and exhibit a “barcode gap” between intraspecific and interspecific genetic divergences (Lahaye et al., 2008; Meyer & Paulay, 2005). We preliminarily evaluated the discrimination ability of the five most commonly used regions, ITS, ITS2, *matK*, *rbcL*, and *psbA‐trnH*, for *Corydalis*. The sequence data for this preliminary evaluation were downloaded from NCBI and have been used in previous molecular phylogenetic studies of Papaveraceae and *Corydalis* (Linden, Fukuhara, Rylander, & Oxelman, 1997; Zhang et al., 2015; Zhang, Wang, & Yang, 2016). The results showed that the *rbcL* and *psbA‐trnH* regions were too well conserved to be able to sufficiently discriminate among species of *Corydalis* (Supporting Information Figures S2 and S3), whereas ITS, ITS2, and *matK *exhibited high rates of nucleotide substitution and a relatively high species discrimination rate.

In this study, we chose three regions (ITS, ITS2, and *matK*) as barcodes for *Corydalis* and performed a systematic comparison of different regions and analytical methods to evaluate primer universality, the DNA barcoding gap and species discrimination efficiency. Our objectives were to (a) determine the performance of DNA barcoding in this taxonomically complex genus, (b) evaluate the species discrimination power of different barcodes, and (c) compare the species resolution rates of different analytical methods. Based on the results obtained in this study, we also discuss the subdivision of *Corydalis* and the potential implications of DNA barcoding in this genus.

## 2 MATERIALS AND METHODS

### 2.1 Sampling strategy

A total of 131 individuals representing 28 *Corydalis* species, including four Pharmacopoeia‐recorded medicinal plants (*C. yanhusuo*,* C. decumbens*,* C. saxicola*, and *C. bungeana*) and their crude drugs (nine leaf specimens of *C. saxicola* and three tuber specimens of each of the other three species), and two outgroups (*Lamprocapnos spectabilis* and *Papaver somniferum*) were used in this study (Table S1). Sequences for 118 individuals of 22 species were obtained de novo, and sequences for 13 individuals were obtained from GenBank. The GenBank sequences are derived from published articles, and we rechecked the sequences through BLAST with conspecifics or closely related species to ensure their correctness. All specimens were identified simultaneously by Professor YingWei Wang (Institute of Botany, Chinese Academy of Sciences), Doctor YaoDong Qi (Institute of Medicinal Plant Development, Chinese Academy of Medical Sciences), and Professor ZhengYu Liu (Chongqing Institute of Medicinal Plant Cultivation). Voucher specimens were deposited at the Institute of Medicinal Plant Development in the Chinese Academy of Medical Sciences. Healthy and fresh leaves of wild plants were collected and dried immediately in silica gel for DNA extraction.

### 2.2 DNA extraction, PCR amplification, and sequencing

Genomic DNA was extracted from silica gel‐dried leaves using a Plant Genomic DNA Kit (Tiangen Biotech, Beijing, China) according to the manufacturer's recommended protocol, which was optimized with slight modifications. These modifications included the addition of more lysis buffer GP1 and cleaning the products one to three times using a nucleus separation liquid until the supernatant layer became lightly colored or colorless.

The PCR amplification of the ITS and *matK* regions was conducted using a Peltier Thermal Cycler PTC2000 (Bio‐Rad) with approximately 30 ng of genomic DNA as the template in 25 μl of 2× Taq PCR MasterMix (Aidlab Biotechnologies, Beijing, China), 1 μl of each primer (2.5 μM, synthesized by Sangon, Shanghai, China), and distilled–deionized water. The primers used for PCR and sequencing and the PCR cycling conditions used in this study are provided in Table 1. For ITS, the primer pair ITSa and ITSb was used for the samples for which the initial PCR amplification or sequencing failed (Table 1, Supporting Information Figure S1). The PCR products were run on a 1.0% agarose gel in 0.5× TBE buffer to assess the success of the amplification and purified with a 1.0% agarose gel using the AXYGEN Gel Recovery Kit (AXYGEN, Hangzhou City, China). The purified PCR products were then sequenced in both directions using the DYEnamic ET Terminator Cycle Sequencing Kit (Amersham Pharmacia Biotech) according to the manufacturer's instructions and analyzed with an ABI 3730XL DNA Sequencer (Applied Biosystems, Foster City, CA, USA).

**Table 1:** Primers, their sequences, and the PCR amplification conditions used in this study

Regions	Primer pairs	Sequence 5ʹ−3ʹ	Thermocycling conditions	Source
ITS	ITS‐5F	GGAAGTAAAAGTCGTAACAAGG	94°C 3 min; [35 cycles: 94°C 60 s, 53°C 90 s, 72°C 90 s]; 72°C 10 min	White, Bruns, Lee, & Taylor, (1990)
	ITS‐4R	CCTTATCATTTAGAGGAAGGAG		
ITSa	ITS‐5F	GGAAGTAAAAGTCGTAACAAGG	94°C 3 min; [35 cycles: 94°C 60 s, 53°C 90 s, 72°C 90 s]; 72°C 10 min	Chen et al. (2010) and by Authors
	ITS‐300R	ATTCACACCAAGTATCGCAT		
ITSb	ITS2‐2F	ATTCACACCAAGTATCGCAT		
	ITS‐4R	CCTTATCATTTAGAGGAAGGAG		
*matK*	3F‐KIM	CGTACAGTACTTTTGTGTTTACGAG	94°C 3 min; [35 cycles: 94°C 60 s, 52°C 60 s, 72°C 90 s]; 72°C 10 min	CBOL Plant Working Group (2009)
	1R‐KIM	ACCCAGTCCATCTGGAAATCTTGGTTC		

### 2.3 Data analysis

All raw sequences, excluding primer regions, were assembled and edited with Condon Code Aligner V 5.1.5 (Condon Code Co., USA). The ITS2 sequences were obtained by removing the conserved 5.8S rRNA and ITS1 sequences of ITS using HMMer, which is based on a hidden Markov model. Sequence alignments for each region were performed using MEGA6.06 (Center for Evolutionary Medicine and Information, USA). Pairwise interspecific and intraspecific genetic distances were calculated based on the Kimura 2‐parameter (K2P) mode. Multiple sequence alignments were concatenated into a single file using Geneious Pro version 4.8.5 (Drummond et al., 2009).

To evaluate the success of species discrimination, the three markers and their possible combinations were analyzed using three widely used methods, namely, tree‐based, similarity‐based, and distance‐based methods. For the tree‐based methods, three different phylogenetic trees, namely, neighbor‐joining (NJ) tree, maximum parsimony (MP) tree, and maximum likelihood (ML) tree, were evaluated to select the most suitable tree. The three trees were constructed with MEGA6.06 according to published protocols for species‐level discrimination within closely related groups (Liu et al., 2012; Tamura, Dudley, Nei, & Kumar, 2007; Yuan et al., 2015). Species discrimination was considered successful if all conspecific individuals formed a single clade. For the similarity‐based method (BLAST), NCBI BLAST 2.2.29+ (Tao, 2010; Yan et al., 2014) was used to build local reference databases, and all the sequences were then queried using the blastn command. Species discrimination was considered successful if all individuals of a species had a top matching hit of only a conspecific individual (Yan et al., 2014). For the distance‐based analysis, we used the K2P‐distance method (Little & Stevenson, 2007). Species discrimination was considered successful if the minimum interspecific K2Pdistance involving a species was larger than the maximum intraspecific distance for that species (CBOL Plant Working Group, 2009; China Plant BOL Group, 2011).

## 3 RESULTS

### 3.1 Barcode universality and sequence characteristics

The separate evaluations of the success rates of PCR amplification and sequencing revealed that the proportions of ITS and *matK* regions were both 100%. With regard to primer universality, ITS exhibited high amplification success with the commonly used primer pair ITS5F/4R, but high‐quality bidirectional sequences from 14 samples (11.0%) could not be generated using this primer pair. Nevertheless, these samples were successfully amplified and sequenced using the primer pair ITSa and ITSb (Tables 1 and 2). Thus, the ITS barcode could be successfully amplified and sequenced from all the samples, and ITS2 sequences were then obtained from the ITS sequences. All *matK* sequences obtained in this study were generated by direct sequencing.

**Table 2:** Evaluation of three DNA barcoding regions

DNA regions	ITS	*matK*	ITS2
Percentage PCR success (%)	100	100	/
Sequencing using a single primer pair (%)	89.0	100	/
No. accessions/total	121	121	121
Sequence length (bp)	519–581	842–858	222–246
Aligned sequence length (bp)	680	883	275
No. variable sites (%)	258 (37.9)	219 (26.3)	123 (44.7)
No. informative sites (%)	194 (28.5)	167 (18.9)	94 (34.2)
No. indel (length in bp)	26 (1–22)	10 (1–12)	9 (1–22)
Intraspecific distance mean (range)	0.0103 (0–0.0317)	0.0016 (0–0.0062)	0.0159 (0–0.0579)
Interspecific distance mean (range)	0.0705 (0.0024–0.1185)	0.0474 (0–0.0878)	0.1026 (0.0014–0.1981)

A total of 393 sequences (ITS, *matK*, and ITS2) for 131 individuals, including 354 sequences from 118 individuals that were newly generated in this study, were available for further analysis. The sequence characteristics of the three regions are summarized in Table 2. The five species with only a single individual and two outgroups (a total of 10 sequences) were excluded from this analysis. Among the three regions, ITS showed the greatest variability in length (519–581 bp) and the highest number of indels (26 indels within a 680‐bp aligned sequence). In contrast, *matK* showed the longest aligned sequence length, the least variability in length (842–858 bp), and the lowest numbers of variable sites (219, 26.3%), informative sites (167, 18.9%) and indels (10 indels within an 883‐bp aligned sequence). ITS2 exhibited the highest numbers of variable sites (123, 44.7%) and informative sites (94, 34.2%; Table 2).

### 3.2 Genetic distance and DNA barcoding gap assessment

**Figure 2:**
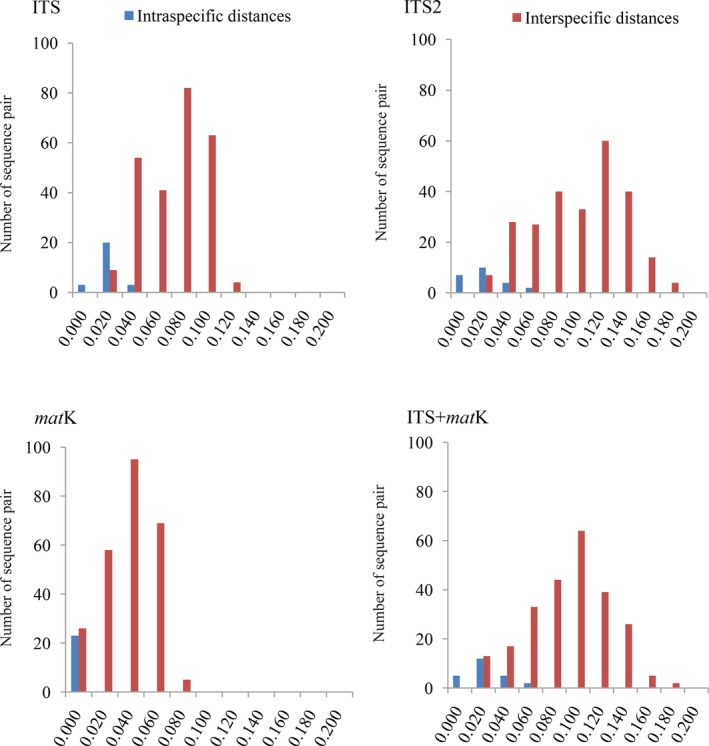
Relative distributions of intraspecific and interspecific K2P distances

Of the three regions, ITS2 exhibited the greatest intraspecific and interspecific distances, followed by ITS, and *matK *showed the lowest values (Table 2). The relative distribution of K2P distances based on single barcodes and barcode combinations is shown in Figure 2.

In general, the mean interspecific distances were greater than the mean intraspecific distances for all three barcodes (Table 2). The rank of the three sequences in terms of mean sequence divergences in *Corydalis* was ITS2>ITS>matK. The barcoding gaps obtained with the single barcodes and barcode combinations are shown in Figure 2.

### 3.3 Comparison of species resolution with different barcodes and their combinations

**Figure 3:**
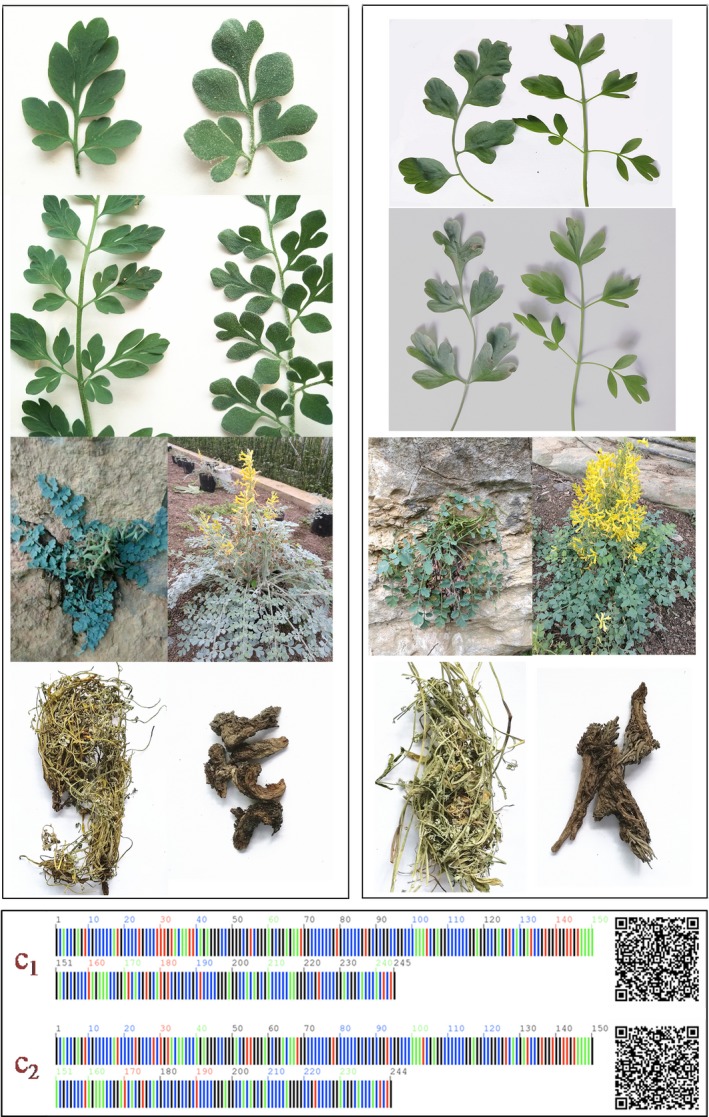
Two closely related medicinal plants *Corydalis tomentella *and *Corydalis saxicola*

**Figure 4:**
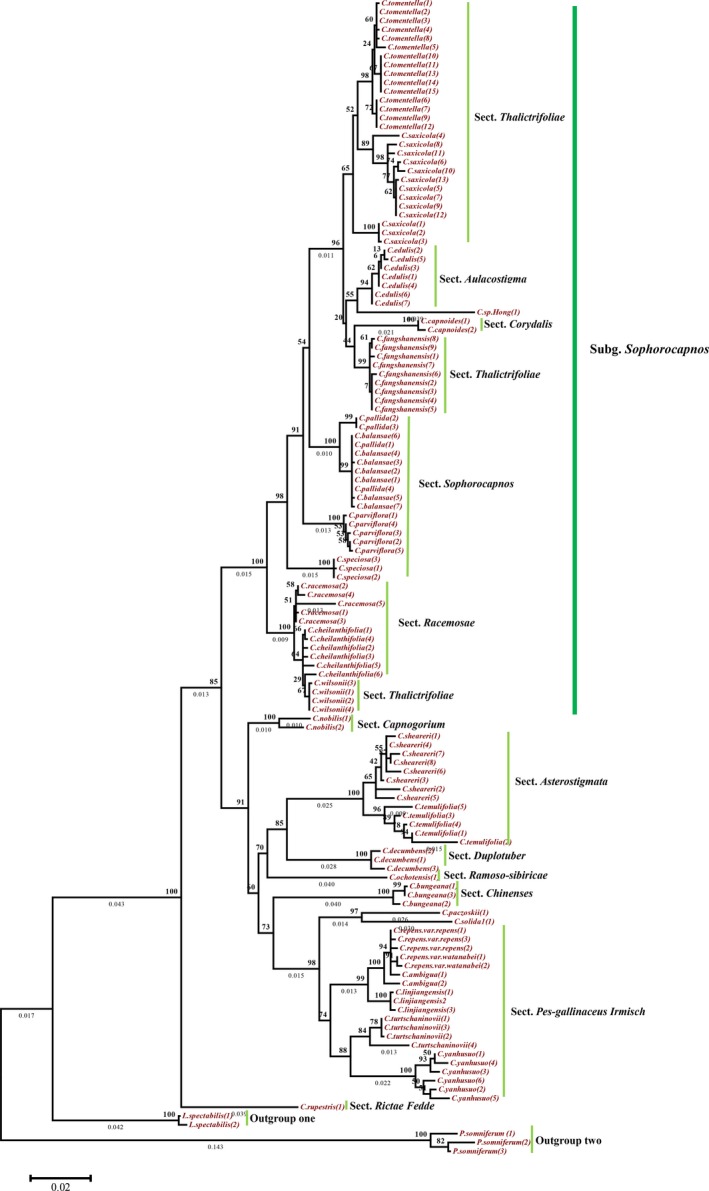
The maximum likelihood tree of 28 *Corydalis *species and two outgroup species of Papaveraceae based on ITS *+ matK* regions

Among the single barcodes, ITS exhibited the highest success rates for the identification of *Corydalis* species, ITS2 showed a lower identification success rate, and *matK* provided the lowest identification success rate (Table 3). Three of the four Pharmacopoeia‐recorded medicinal plants (*C. yanhusuo*,* C. decumbens*, and *C. bungeana*) and their crude drugs were identified successfully using the ITS and ITS2 regions (Figure 4). The Pharmacopoeia‐recorded species *C. saxicola* and its close relative *C. tomentella* was discriminated using the QR code (two‐dimensional code) of the ITS2 region (Figure 3).

**Table 3:** The comparative analysis of different analytical methods for species resolution

Species resolution (%) of the potential barcodes					
Method	ITS	*mat*K	ITS2	ITS + matK	ITS2 + *mat*K
BLAST	65.2	56.5	60.9	69.6	60.9
K2P‐distance	56.5	52.2	52.2	65.2	65.2
NJ tree	65.2	47.8	60.9	65.2	65.2
ML tree	60.9	52.2	56.5	60.9	60.9
MP tree	52.2	47.8	52.2	60.9	60.9

Among the barcode combinations, ITS + matK provided a higher discrimination success rate than ITS2 + *matK*. In addition, the barcode combinations exhibited a higher discrimination success rate than any single barcode (Table 3).

Among all single barcodes and their combinations used in this study, ITS + matK provided the highest identification success rate, and *matK* alone provided the lowest identification success rate (Table 3).

### 3.4 Comparison of different analytical methods for species resolution

The species discrimination ability depends on the analytical method used. A comparison of different analytical methods using data obtained with a single barcode revealed that the BLAST method yielded the highest discrimination success rate (Table 3 and S2‐S6). The barcode combinations yielded different results. When applied to data obtained from the combination ITS + matK, BLAST provided the highest identification success rate, but when applied to data obtained with ITS2 + *matK*, the NJ tree‐ and K2P distance‐based methods showed the highest identification success rate (Table 3).

The comparisons of different analytical methods using data obtained with all the single barcodes and their combinations used in this study showed that BLAST tended to provide the highest discrimination success for all barcodes with the exception of ITS2 + *matK*. The results obtained using the NJ tree‐based method were similar to but slightly better than those obtained using the K2P distance‐based method (Table 3). Overall, regardless of the method used, the barcode combinations nearly always resulted in improved species resolution, and in this study, the barcode combination ITS + matK (69.6%) with the BLAST method provided the highest species resolution.

### 3.5 Phylogenetic analyses of chloroplast and nuclear DNA regions

The ML tree constructed using the data obtained with ITS + matK recovered *Corydalis* as a monophyletic group. *C. rupestris *was strongly supported as the basal taxon that diverged first, and two major clades were then recognized (Figure 4). The first clade included two subclades: four species of sect. *Thalictrifoliae*, one species of sect. *Aulacostigma*, three species of sect. *Sophorocapnos*, and one species of sect. *Corydalis* formed one subclade, and two species of sect. *Racemosae* and one species of sect. *Thalictrifoliae* formed the other subclade. The second clade included three subclades: one of these subclades consisted of five species of sect. *Pes‐gallinaceus Irmisch* and one species of sect. *Chinenses*; the second subclade consisted of one species of sect. *Capnogorium*; and the third subclade included two species of sect. *Asterostigma*, one species of sect. *Duplotuber* and one species of sect. *Ramoso‐sibiricae* (Figure 4).

The ML tree constructed using the data obtained with ITS was slightly distinct from that constructed using the data obtained with ITS + matK. The ITS‐based ML tree recovered *Corydalis* as a monophyletic group and revealed two well‐supported clades. The division of the two clades into two subclades was weakly supported. Furthermore, compared with the ITS + matK‐based tree, the phylogenetic positions of some species were changed in the ITS‐based tree. Specifically, *C. rupestris* and *C. capnoides *formed a subclade that diverged from the first clade, and *C. decumbens* and *C. ochotensis *formed a clade that diverged from the second subclade (Supporting Information Figure S4).

The ML tree constructed using the data obtained with *matK* was similar to the ITS + matK‐based tree. It also recovered *Corydalis* as a monophyletic group, *C. rupestris *was strongly supported as the basal taxon and diverged first, and the remaining species formed two well‐supported clades. The species composition of the first clade was consistent with that found with the ITS + matK‐based tree. However, the second clade did not divide into three subclades as in the ITS + matK‐based tree; instead, *C. nobilis* of sect. *Capnogorium *formed its own subclade that diverged first as a sister subclade to the rest of the clade, and the second subclade was then further divided into two clades (Supporting Information Figure S5).

## 4 DISCUSSION

### 4.1 Evaluation of DNA barcodes for Corydalis

*Corydalis *is one of the most taxonomically complicated plant genera, and the discrimination of species within this genus has always been recognized as a great challenge. Jiang et al. (2018) used DNA barcoding to identify two herbal species of *Corydalis* in the Pharmacopoeia of China based on 57 samples of 14 species, and their results showed that DNA barcoding can be used as an effective method for the identification of medicinal species belonging to *Corydalis*. In this study, a total of 131 individuals of 28 *Corydalis* species and two outgroups were assessed, including 21 species that were not previously evaluated. The specimens included representatives of all three subgenera (Linden et al., 1997) and all four herbal species of *Corydalis* in the Pharmacopoeia of China. In view of the widespread existence of infraspecific polymorphisms in *Corydalis *(Wu, 1996), we analyzed a large number of samples of each species with high morphological variation (up to 15 samples of a single species), which contributed to the determination of authentic phylogenetic relationships. Based on these data, we performed a systematic comparison of different loci and analytical methods to select appropriate barcodes and analytical methods for *Corydalis*.

The evaluation of different analytical methods showed that BLAST exhibited the highest species discrimination ability. The superior performance of the BLAST approach compared with other methods has been observed in several previous studies (Chen et al., 2010; Kool et al., 2012; Li et al., 2011; Van Velzen et al., 2012; Yan et al., 2014). The species discrimination power of an approach is related to the theory and algorithm used by the approach. BLAST often shows significantly higher identification rates than other approaches (Chen et al., 2010; Li et al., 2011; Sandionigi et al., 2012), and it appears to be the best choice for the identification of *Corydalis* species. The NJ tree usually shows low species resolution, which significantly reduces its usefulness (Liu et al., 2012; Van Velzen et al., 2012; Yan et al., 2014), and in this study, the NJ tree provided a lower species resolution than BLAST. However, because of its advantages of faster speed and a more intuitive display of genetic relationships, which facilitates understanding and analysis, we still advocate that the NJ tree‐based method is useful for the discrimination of *Corydalis* species.

The comparison of different barcodes indicated that ITS exhibited the highest species resolution (65.2%) among all three single barcodes evaluated in this study. The resolution level obtained in this study is equivalent to the average level (67.2%) observed in a large dataset of 5,583 samples representing 1,349 species in 141 genera (Li et al., 2011). Compared with the species resolution obtained with ITS for other large genera, such as *Rhododendron *(12.2%, 15.3%), *Angelica *(73.9%), *Pedicularis *(86.2%), and *Primula *(88.2%) (Li et al., 2011; Yan et al., 2014; Yuan et al., 2015), that obtained in this study corresponds to a medium level of identification efficiency. However, a previous study showed that the ITS region is not suitable for the molecular analysis of *Corydalis* due to a low PCR amplification and sequencing success rates (61.9% and 28.6%, respectively) and that *matK* provides the highest species resolution (100%) and can thus be considered an ideal barcode for *Corydalis* (Jiang et al., 2018). In this study, we initially used the universal primer pair ITS5F/4R and obtained low PCR amplification and sequencing efficiency. Therefore, we designed two pairs of primers to perform a fractional amplification of the full‐length sequence of ITS and thus obtain a complete ITS sequence (Table 1, Supporting Information Figure S1), and our resulting PCR amplification and sequencing success rates were both 100%. The ITS and *matK* regions were then evaluated using 131 samples representing 28 species of *Corydalis*, which included 23 species with more than three samples and 16 species that were not included in previous studies (two species are herbal species in the Pharmacopoeia of China) (Jiang et al., 2018). Our results showed that ITS exhibited a higher species resolution (65.2%) than *matK* (56.5%)，15 of the 23 species could be successfully identified by ITS, 13 of the 23 species could be successfully identified by *matK*, and one herbal species in the Pharmacopoeia of China could not be successfully identified by *matK*. The high species resolution of DNA barcoding is likely due to the relatively small sample size and wide taxonomic sampling employed (Li et al., 2011; Yan et al., 2014), and the species identification resolution usually decreases with increases in the sample size. Thus, sufficient sampling for a taxon‐based DNA barcoding study is a pivotal issue that should be considered (Yan et al., 2014). Based on the highest species resolution obtained with a single barcode in this study and the successful optimization of PCR amplification and sequencing methods, we considered ITS to be the most appropriate barcode for the discrimination of species belonging to the genus *Corydalis*.

The combination of DNA barcodes usually improves species identification (CBOL Plant Working Group, 2009; Li et al., 2011; Yan et al., 2014; Yuan et al., 2015). In this study, any combination of the barcodes yielded higher discrimination rates. Although *matK* provided the lowest species identification ability when used alone, combinations including *matK* exhibited significantly increased discrimination power. Thus, *matK* can be used as an additional barcode for *Corydalis*, and ITS + matK was identified as the best barcode combination for *Corydalis*.

### 4.2 Phylogenetic analysis of Corydalis based on DNA barcoding

Wu (1996) first classified this genus into 40 sections and two subgenera, subg. *Corydalis* and subg. *Pistolochia*, based on morphological characteristics and geographical distributions. Linden (1997) claimed that this genus should be divided into 25 sections and three subgenera, namely, subg. *Chremnocapnos*, subg*. Sophorocapnos*, and subg. *Corydalis*, based on morphological characteristics and the *rps*16 locus. In recent years, with the continuous uncovering of new evidence and the accumulation of new taxa, the section division proposed by Linden has tended to be consistent with the structure proposed by Wu. Based on molecular systematics (*rps*16 and *matK*) and palynology, Wang (2006) advocated that the most appropriate division of *Corydalis* is into five subgenera, namely, subg. *Chremnocapnos*, subg. *Sophorocapnos*, subg. *Corydalis*, subg. *Rapiferae*, and subg. *Fasciculatae*. In all the trees constructed in this study, the genus *Corydalis *tended to be divided into two major clades. The first major clade included sect. *Thalictrifoliae*, sect. *Sophorocapnos*, sect. *Racemosae*, sect. *Aulacostigma*, and sect. *Corydalis*, and the first four of these sections were classified into Wu's subg. *Corydalis *(Wu, 1996), Linden's subg. *Sophorocapnos *(Linden et al., 1995), and Wang's subg. *Sophorocapnos* (Wang, 2006), respectively. However, subg. *Sophorocapnos *(in Linden's and Wang's classifications) can be included in subg. *Corydalis *(in Wu's classification). Thus, all the evidence supports the classification of sect. *Thalictrifoliae*, sect. *Sophorocapnos*, sect. *Racemosae*, and sect. *Aulacostigma *into one subgenus. In addition, this study constitutes the first evaluation of sect. *Corydalis*, and all the phylogenetic trees constructed in this study support the inclusion of sect. *Corydalis *in this subgenus.

In the second major clade, the further divisions created by the ML tree using different loci exhibited a number of discrepancies. The *matK*‐ and ITS + matK‐based trees were strongly supported, whereas the ITS‐based tree was weakly supported; therefore, we will mainly refer to the ITS + matK‐based tree. The second major clade tended to be divided into two larger subclades and one smaller subclade. Sections *Pes‐gallinaceus Irmisch* and *Chinenses* formed the first larger subclade; sections *Asterostigma*, *Duplotuber*, and *Ramoso‐sibiricae* formed the second larger subclade; and sect. *Capnogorium* alone formed the smaller subclade. In Wang's division of subgenera (Wang, 2006), subg. *Sophorocapnos *was recognized in the first major clade, but the remaining subgenera in the second major clade could not be recognized, and our results, therefore, do not support Wang's division. In the second larger subclade, sect. *Ramoso‐sibiricae* and sect. *Asterostigma* are divided into subg. *Corydalis*, as in Wu's classification (Wu, 1996). Although our results support the division of this genus into two major subgenera, we do not agree with the species coverage of subgenera in Wu's division.

Overall, Wu (1996) classified *Corydalis* into two subgenera based on morphological characteristics and geographical distributions. Linden (1997) claimed that this genus was divided into three subgenera based on morphological characteristics and the *rps*16 locus. Using molecular systematics (*rps*16 and *matK*) and palynology, Wang (2006) advocated that the most appropriate division of *Corydalis* was five subgenera. Our molecular evidence is consistent with Linden's proposal, and we tended to divide this genus into three subgenera. In the subgenus, four sections, namely, sect. *Thalictrifoliae*, sect. *Sophorocapnos*, sect. *Racemosae*, and sect. *Aulacostigma*, which are recognized in the division proposed by Linden (1997) and Wang (2006), have been classified within subg. *Sophorocapnos*, and our molecular results support this view. Furthermore, this study provides the first molecular analysis of sect. *Corydalis*, and its inclusion in subg. *Sophorocapnos* is strongly supported by our phylogenetic analysis. Therefore, we suggest that sect. *Thalictrifoliae*, sect. *Sophorocapnos*, sect. *Racemosae*, sect. *Aulacostigma*, and sect. *Corydalis *should be classified as one subgenus. Of course, more molecular and morphological evidence will be required to test this finding. Our current results based on molecular data are expected to provide some reference for future research.

### 4.3 Potential utility of DNA barcoding in Corydalis

The genus *Corydalis* is an important group of Papaveraceae. *Corydalis* plants contain a variety of alkaloid components that possess potent pharmacological effects, including antinociceptive (Wang et al., 2016), anticardiovascular (Hung & Wu, 2016), antitumor (Gao, He, Li, & Wang, 2009), antidepressive (Du et al., 2018), hepatoprotective (Wu et al., 2017), acetylcholinesterase inhibitory, and butyrylcholinesterase inhibitory effects (Orhan, Şener, Choudhary, & Khalid, 2004). More than 30 species are used in folk medicine or are recorded in the Pharmacopoeia (Pharmacopoeia Commission of People's Republic of China, 2015; Sang, 2002), and these species are often closely related. To ensure the safety, efficacy, and legality of these medicines, accurate identification is essential. The authentication of materia medica is time‐consuming and knowledge‐intensive because they are always presented as crude drugs or decoctions, which are air‐dried or processed via various methods and thus exhibit modified morphological and anatomical features (Yuan et al., 2015). DNA technology exhibits an outstanding advantage in that it is not affected by morphological characteristics, developmental stage, environmental factors, or harvesting period (Heubl, 2010). Since Chen et al. (2010) proposed ITS2 as a standard DNA barcode for the identification of medicinal plants, DNA barcoding has been increasingly used in the authentication of medicinal plants and materia medica (Xin et al., 2012; Yuan et al., 2015). In this study, three Pharmacopoeia‐recorded medicinal plants and their crude drugs were identified successfully, and another Pharmacopoeia‐recorded species was discriminated from closely related plants. Based on the good performance of DNA barcoding in *Corydalis*, it can be used as a potential tool for the authentication of the medicinal plants and materia medica belonging to this genus.

The short‐region DNA barcodes showed a relatively high species discrimination power for *Corydalis* species in this study; however, 30.4% of the species, most of which are closely related, could not be identified successfully. Recent barcoding studies have placed high emphasis on the use of whole‐chloroplast genome sequences, which are now more readily available due to improvements in sequencing technologies (Li et al., 2015). The whole‐chloroplast genome is termed a “super‐barcode” and provides more abundant informative sites for species identification. These super‐barcodes exhibit higher discrimination power for closely related species and have been applied for the identification of species in various taxa, such as the genera *Fritillaria* (Li, Zhang, Yang, & Lv, 2018), *Epipremnum *(Tian, Han, Chen, & Wang, 2018), and *Papaver *(Zhou et al., 2017). The use of a super‐barcodes is a good option for the identification of closely related *Corydalis* species and obtaining improved species discrimination power. The use of data inferred from DNA barcodes and whole‐chloroplast genomes, along with data on morphological characteristics and geographical distributions, might result in more precise species discrimination and resolve phylogenetic disputes, which will aid the reconstruction of a more integrated taxonomic system of this taxonomically complicated genus.

## CONFLICT OF INTEREST

The authors declare that the research was conducted in the absence of any commercial or financial relationships that could be construed as a potential conflict of interest.

## AUTHOR CONTRIBUTIONS

The study was conceived by FR, JS, sample collection was performed by FR, YW, YQ, data generation was carried out by YW, data were analyzed by ZX, YL, JZ, and TX, the manuscript was written by FR, JS.

## Data Availability

DNA sequences: GenBank accessions MH349216‐MH349333.

## References

[ece34886-bib-0001] Austerlitz, F. , David, O. , Schaeffer, B. , Bleakley, K. , Olteanu, M. , Leblois, R. , … Laredo, C. (2009). DNA barcode analysis: A comparison of phylogenetic and statistical classification methods. BMC Bioinformatics, 10, S10 10.1186/1471-2105-10-S14-S10. PMC2775147 19900297

[ece34886-bib-0002] Barco, A. , Raupach, M. J. , Laakmann, S. , Neumann, H. , & Knebelsberger, T. (2016). Identification of North Sea molluscs with DNA barcoding. Molecular Ecology Resources, 16, 288–297. 10.1111/1755-0998.12440 26095230

[ece34886-bib-0003] CBOL Plant Working Group . (2009). A DNA barcode for land plants. Proceedings of the National Academy of Sciences of the United States of America, 106, 12794–12797. 10.1073/pnas.0905845106 19666622 PMC2722355

[ece34886-bib-0004] Chen, S. L. , Yao, H. , Han, J. P. , Liu, C. , Song, J. , Shi, L. , … Leon, C. (2010). Validation of the ITS2 region as anovel DNA barcode for identifying medicinal plant species. PLoS ONE, 5, e8613 10.1371/journal.pone.0008613 20062805 PMC2799520

[ece34886-bib-0005] Chen, Z. D. , Lu, A. M. , Zhang, S. Z. , Wang, Q.‐F. , Liu, Z.‐J. , Li, D.‐Z. , … China Phylogeny Consortium . (2016). The tree of life: China project. Journal of Systematics & Evolution, 54(4), 273–276.

[ece34886-bib-0006] China Plant BOL Group (2011). Comparative analysis of a large dataset indicates that ITS should be incorporated into the core barcode for seed plants. Proceedings of the National Academy of Sciences of the United States of America, 108, 19641–19646. 10.1073/pnas.1104551108. 22100737 10.1073/pnas.1104551108 PMC3241788

[ece34886-bib-0007] Drummond, A. J. , Ashton, B. , Cheung, M. , Heled, J. , Kearse, M. , Moir, R. , … Wilson, A. (2009). *Geneious v4.7* Retrieved from http://www.geneious.com.

[ece34886-bib-0008] Du, W. , Jin, L. , Li, L. , Wang, W. , Zeng, S. , Jiang, H. , & Zhou, H. (2018). Development and Validation of a HPLC‐ESI‐MS/MS method for simultaneous quantification of fourteen alkaloids in mouse plasma after oral administration of the extract of *Corydalis* yanhusuo tuber: Application to pharmacokinetic study. Molecules, 2018(23), 714 10.3390/molecules23040714 PMC6017933 29561801

[ece34886-bib-0009] Frezal, L. , & Leblois, R. (2008). Four years of DNA barcoding: Current advances and prospects. Infection, Genetics and Evolution, 8, 727–736. 10.1016/j.meegid.2008.05.005 18573351

[ece34886-bib-0010] Gao, J. L. , He, T. C. , Li, Y. B. , & Wang, Y. T. (2009). A traditional chinese medicine formulation consisting of rhizoma *corydalis* and rhizoma curcumae exerts synergistic anti‐tumor activity. Oncology Reports, 22(5), 1077–1083. 19787224 10.3892/or_00000539

[ece34886-bib-0011] Han, J. P. , Zhu, Y. , Chen, X. , Liao, B. , Yao, H. , Song, J. , … Meng, F. (2013). The short ITS2 sequence serves as an efficient taxonomic sequence tag in comparison with the full‐length ITS. BioMed Research International, 2013(5), 1–7. 10.1155/2013/741476 PMC3581084 23484151

[ece34886-bib-0012] Hebert, P. D. , Cywinska, A. , Ball, S. L. , & deWaard, J. R. (2003). Biological identifications through DNA barcodes. Proceedings. Biological sciences/The RoyalSociety, 270, 313–321. 10.1098/rspb.2002.2218 PMC1691236 12614582

[ece34886-bib-0013] Heubl, G. (2010). New aspects of DNA‐based authentication of Chinese medicinal plants by molecular biological techniques. Planta Medica, 76, 1963–1974. 10.1055/s-0030-1250519 21058240

[ece34886-bib-0014] Hollingsworth, P. M. , Graham, S. W. , & Little, D. P. (2011). Choosing and using a plant DNA barcode. PLoS One, 6, e19254 10.1371/journal.pone.0019254. 21637336 10.1371/journal.pone.0019254 PMC3102656

[ece34886-bib-0015] Huemer, P. , Karsholt, O. , & Mutanen, M. (2014). DNA barcoding as a screening tool for cryptic diversity: An example from Caryocolum, with description of a new species (Lepidoptera, Gelechiidae). ZooKeys, 404, 91–111. 10.3897/zookeys.404.7234 PMC4023261 24843272

[ece34886-bib-0016] Hung, H. Y. , & Wu, T. S. (2016). Recent progress on the traditional Chinese medicines that regulate the blood. Journal of Food & Drug Analysis, 24(2), 221–238. 10.1016/j.jfda.2015.10.009 28911575 PMC9339571

[ece34886-bib-0017] Ji, Y. , Ashton, L. , Pedley, S. M. , Edwards, D. P. , Tang, Y. , Nakamura, A. , … Yu, D. W. (2013). Reliable, verifiable and efficient monitoring of biodiversity via meta barcoding. Ecology Letters, 16, 1245–1257. 10.1111/ele.12162 23910579

[ece34886-bib-0018] Jiang, L. , Li, M. , Zhao, F. X. , Chu, S. S. , Zha, L. P. , Xu, T. , … Zhang, W. (2018). Molecular identification and taxonomic implication of herbal species in genus Corydalis (Papaveraceae). Molecules, 23(6), 1393 10.3390/molecules23061393 PMC6100380 29890665

[ece34886-bib-0019] Kool, A. , de Boer, H. J. , Krüger, A. , Rydberg, A. , Abbad, A. , Björk, L. , & Martin, G. (2012). Molecular identification of commercialized medicinal plants in Southern Morocco. PLoS ONE, 7, e39459 10.1371/journal.pone.0039459 22761800 PMC3384669

[ece34886-bib-0020] Kress, W. J. , Wurdack, K. J. , Zimmer, E. A. , Weigt, L. A. , & Janzen, D. H. (2005). Use of DNA barcodes to identify flowering plants. Proceedings of the National Academy of Sciences of the United States of America, 102, 8369–8374. 10.1073/pnas.0503123102 15928076 PMC1142120

[ece34886-bib-0021] Lahaye, R. , Van der Bank, M. , Bogarin, D. , Warner, J. , Pupulin, F. , Gigot, G. , … Savolainen, V. (2008). DNA barcoding the floras of biodiversity hotspots. Proceedings of the National Academy of Sciences of the United States of America, 105, 2923–2928. 10.1073/pnas.0709936105 18258745 PMC2268561

[ece34886-bib-0022] Li, D. Z. , Gao, L. M. , Li, H. T. , Wang, H. , Ge, X. J. , Liu, J. Q. , … Duan, G. W. (2011). Comparative analysis of a large dataset indicates that internal transcribed spacer (ITS) should be incorporated into the core barcode for seed plants. Proceedings of the National Academy of Sciences of the United States of America, 108, 19641–19646. 10.1073/pnas.1104551108 22100737 PMC3241788

[ece34886-bib-0023] Li, X. , Yang, Y. , Henry, R. J. , Rossetto, M. , Wang, Y. , & Chen, S. (2015). Plant DNA barcoding: From gene to genome. Biological Reviews, 90, 157–166. 10.1111/brv.12104 24666563

[ece34886-bib-0024] Li, Y. , Zhang, Z. , Yang, J. , & Lv, G. (2018). Complete chloroplast genome of seven *Fritillaria* species, variable DNA markers identification and phylogenetic relationships within the genus. PLoS ONE, 13(3), e0194613 10.1371/journal.pone.0194613 29543905 PMC5854438

[ece34886-bib-0025] Linden, M. , Fukuhara, T. , & Axberg, T. (1995). Phylogeny of *Corydalis*, ITS and morphology. Plant Systematics and Evolution ‐ Supplementa, 9, 183e188.

[ece34886-bib-0026] Linden, M. , Fukuhara, T. , Rylander, J. , & Oxelman, B. (1997). Phylogeny and classification of Fumariaceae, with emphasis on Dicentra s. l., based on the chloroplast gene rps16 intron. Plant Systematics and Evolution, 206, 411e420.

[ece34886-bib-0027] Little, D. P. , & Stevenson, D. W. (2007). A comparison of algorithms for the identification of specimens using DNA barcodes: Examples from gymnosperms. Cladistics, 23, 1–21. 10.1111/j.1096-0031.2006.00126.x 34905841

[ece34886-bib-0028] Liu , J. , Möller , M. , Gao , L. M. , Zhang , D. Q. , & Li , D. Z. (2011). DNA barcoding for the discrimination of Eurasian yews (Taxus L., Taxaceae) and the discovery of cryptic species. Molecular Ecology Resources, 11, 89–100. 10.1111/j.1755-0998.2010.02907.x 21429104

[ece34886-bib-0029] Liu, J. , Provan, J. , Gao, L. M. , & Li, D. Z. (2012). Sampling strategy and potential utility of indels for DNA barcoding of closely related plant species: A case study in Taxus. International Journal of Molecular Sciences, 13, 8740–8751. 10.3390/ijms13078740 22942731 PMC3430262

[ece34886-bib-0030] Meyer, C. P. , & Paulay, G. (2005). DNA barcoding: Error rates based on comprehensive sampling. PLoS Biology, 3, e422 10.1371/journal.pbio.0030422 16336051 PMC1287506

[ece34886-bib-0031] Orhan, I. , Şener, B. , Choudhary, M. I. , & Khalid, A. (2004). Acetylcholinesterase and butyrylcholinesterase inhibitory activity of some turkish medicinal plants. Journal of Ethnopharmacology, 91(1), 57–60. 10.1016/j.jep.2003.11.016 15036468

[ece34886-bib-0032] Pharmacopoeia Commission of People's Republic of China . (2015). Pharmacopoeia of People's Republic of China. Beijing: China Medical Science Press.

[ece34886-bib-0033] Sandionigi, A. , Galimberti, A. , Labra, M. , Ferri, E. , Panunzi, E. , & De Mattia, F. (2012). Analytical approaches for DNA barcoding data‐how to find a way for plants. Plant Biosystems‐An International Journal Dealing with All Aspects of Plant Biology, 146, 805–813.

[ece34886-bib-0034] Sang, T. (2002). Utility of low‐copy nuclear gene sequences in plant phylogenetics. Critical Reviews in Biochemistry and Molecular Biology, 37, 121–147. 12139440 10.1080/10409230290771474

[ece34886-bib-0035] Sucher, N. J. , & Carles, M. C. (2008). Genome‐based approaches to the authentication of medicinal plants. Planta Medica, 74, 603–623. 10.1055/s-2008-1074517 18449847

[ece34886-bib-0036] Taberlet, P. , Coissac, E. , Pompanon, F. , Brochmann, C. , & Willerslev, E. (2012). Towards next‐generation biodiversity assessment using DNA metabarcoding. Molecular Ecology, 21, 2045–2050. 10.1111/j.1365-294X.2012.05470.x 22486824

[ece34886-bib-0037] Tamura, K. , Dudley, J. , Nei, M. , & Kumar, S. (2007). MEGA4: Molecular evolutionary genetics analysis (MEGA) software version 4.0. Molecular Biology and Evolution, 24, 1596–1599. 10.1093/molbev/msm092 17488738

[ece34886-bib-0038] Tao, T. (2010). Standalone BLAST Setup for Windows PC. Retrieved from http://www.ncbi.nlm.nih.gov/books/NBK52637/.

[ece34886-bib-0039] Tian, N. , Han, L. , Chen, C. , & Wang, Z. (2018). The complete chloroplast genome sequence of *Epipremnum aureum* and its comparative analysis among eight Araceae species. PLoS ONE, 13(3), e0192956 10.1371/journal.pone.0192956 29529038 PMC5846728

[ece34886-bib-0040] Van Velzen, R. , Weitschek, E. , Felici, G. , & Bakker, F. T. (2012). DNA barcoding of recently diverged species: Relative performance of matching methods. PLoS ONE, 7, e30490 10.1371/journal.pone.0030490 22272356 PMC3260286

[ece34886-bib-0041] Wang, L. , Zhang, Y. , Wang, Z. , Gong, N. , Kweon, T. D. , Vo, B. , … Civelli, O. (2016). The antinociceptive properties of the *Corydalis* *yanhusuo* Extract. PLoS ONE, 11(9), e0162875 10.1371/journal.pone.0162875 27622550 PMC5021270

[ece34886-bib-0042] Wang, Y. W. (2006). Study on the phylogenetic of *Corydalis*. Institute of Plant Science, Graduate University of Chinese Academy of Sciences.

[ece34886-bib-0043] White, T. J. , Bruns, T. , Lee, S. , & Taylor, J. (1990). PCR protocols: A guide to methods and applications (pp. 315–322). New York, NY: Academic Press.

[ece34886-bib-0044] Wu, F. , Zheng, H. , Yang, Z. T. , Cheng, B. , Wu, J. X. , Liu, X. W. , … Su, Z. H. (2017). Urinary metabonomics study of the hepatoprotective effects of total alkaloids from *Corydalis* saxicola bunting on carbon tetrachloride‐induced chronic hepatotoxicity in rats using 1hnmr analysis. Journal of Pharmaceutical and Biomedical Analysis, 140, 199–209. 10.1016/j.jpba.2017.03.031. 28363136

[ece34886-bib-0045] Wu, Z. Y. (1996). The systematic evolution of *Corydalis* in relation to florogenesis and floristic regionalization in the world. Acta Botanica Yunnanica, 18, 241–267.

[ece34886-bib-0046] Xin, T. Y. , Yao, H. , Luo, K. , Xiang, L. , Ma, X. C. , Han, J. P. , … Chen, S. L. (2012). Stability and accuracy of the identification of notopterygii rhizoma et radix using the its/its2 barcodes. Acta Pharmaceutica Sinica, 47(8), 1098–1105. 23162910

[ece34886-bib-0047] Yan, L. J. , Liu, J. , Möller, M. , Zhang, L. , Zhang, X. M. , Li, D. Z. , & Gao, L. M. (2014). DNA barcoding of *Rhododendron* (Ericaceae), the largest Chinese plant genus in biodiversity hotspots of the Himalaya‐Hengduan Mountains. Molecular Ecology Resources, 15, 932–944. 10.1111/1755-0998.12353 25469426

[ece34886-bib-0048] Yao, H. , Song, J. , Liu, C. , Luo, K. , Han, J. , Li, Y. , … Chen, S. (2010). Use of ITS2 region as the universal DNA barcode for plants and animals. PLoS ONE, 5, e13102 10.1371/journal.pone.0013102 20957043 PMC2948509

[ece34886-bib-0049] Yuan, Q. J. , Zhang, B. , Jiang, D. , Zhang, W. J. , Lin, T. Y. , Wang, N. H. , … Huang, L. Q. (2015). Identification of species and materia medica within Angelica L. (Umbelliferae) based on phylogeny inferred from DNA barcodes. Molecular Ecology Resources, 15, 358–371. 10.1111/1755-0998.12296 24961287 PMC4344822

[ece34886-bib-0050] Zemlak, T. S. , Ward, R. D. , Connell, A. D. , Holmes, B. H. , & Hebert, P. D. (2009). DNA barcoding reveals overlooked marine fishes. Molecular Ecology Resources, 9, 237–242. 10.1111/j.1755-0998.2009.02649.x 21564983

[ece34886-bib-0051] Zhang, M. L. , Su, Z. Y. , & Liden, M. (2008). Corydalis DC In: Wu Z. Y., Raven P. H., & Hong D. Y. (eds.), Flora of China, Volume 7, Menispermaceae through Capparaceae (pp. 295–428). Beijing, China: Science Press; St. Louis, MO: Missouri Botanical Garden Press.

[ece34886-bib-0052] Zhang, Z. X. , Niu, H. Y. , Guo, X. , Wang, D. , & Eaton, W. D. (2015). Genetic diversity and genetic structure of *Corydalis* tomentella, franch. (papaveraceae), an endangered herb species from central china. Biochemical Systematics & Ecology, 63(346), 27–33.

[ece34886-bib-0053] Zhang, Z. X. , Wang, D. , & Yang, X. (2016). The taxonomic position of Corydalis parviflora Su & Liden (Papaveraceae), a genetically distinct species: Evidence from cpDNA and nDNA sequences. Biochemical Systematics and Ecology, 67, 134–141.

[ece34886-bib-0054] Zhou, J. , Chen, X. , Cui, Y. , Sun, W. , Li, Y. , Wang, Y. , … Yao, H. (2017). Molecular structure and phylogenetic analyses of complete chloroplast genomes of two *aristolochia* medicinal species. International Journal of Molecular Sciences, 18(9), 1839 10.3390/ijms18091839 PMC5618488 28837061

