# Supplementary material for: DNA barcoding of Corydalis, the most taxonomically complicated genus of Papaveraceae
Source: Ecol Evol. 2019 Jan 21;9(4):1934–45. doi: 10.1002/ece3.4886 (PMC6392370; doi:10.1002/ece3.4886)
Supplement: Supplementary file 1 [file ECE3-9-1934-s001.docx]

**Ecology and Evolution**

SUPPORTING INFORMATION

**DNA barcoding of *Corydalis*, the most taxonomically complicated genus of Papaveraceae**

**Feng-Ming REN^1,2^, Ying-Wei Wang^3^, Zhi-Chao Xu^1^, Ying Li^1^, Tian-Yi Xin^1^, Jian-Guo Zhou^1^, Yao-Dong Qi^1^, Xue-Ping Wei^1^, Hui Yao^1^ and Jing-Yuan Song^1^**

**ITS-5F**

**ITS-4R**

**ITS2**

**ITS1**

**5.8S**

**ITS2-2F**

**ITS-300R**

**FIGURE S1** The PCR amplification schematic diagram of two primer pairs ITSa and ITSb. The solid arrowhead represents the primer ITSa, and the hollow arrowhead represents the primer ITSb.

**FIGURE S2** The NJ tree of 28 sequences from 10 *Corydalis* species based on *psbA-trnH* region. The preliminary evaluation of *psbA-trnH* for ability to discriminate *Corydalis*

species*,* the sequence data were downloaded from NCBI. Only four of ten species were identified successfully by *psbA-trnH* region.

**FIGURE S3** The NJ tree of 14 sequences from 8 *Corydalis* species based on *rbcL* region. The preliminary evaluation of *rbcL* for ability to discriminate *Corydalis* species, the sequence data were downloaded from NCBI. Only three of eight species were identified successfully by *rbcL* region.

**FIGURE S4** The maximum likelihood tree of 28 *Corydalis* species and two outgroup species of Papaveraceae based on ITS region

**FIGURE S5** The maximum likelihood tree of 28 *Corydalis* species and two outgroup species of Papaveraceae based on *matK* region

**FIGURE S6** The NJ tree of 23 *Corydalis* species based on ITS region

**FIGURE S7** The ML tree of 23 *Corydalis* species based on ITS region

**

**FIGURE S8** The MP tree of 23 *Corydalis* species based on ITS region

**FIGURE S9** The NJ tree of 23 *Corydalis* species based on *matK* region

**FIGURE S10** The ML tree of 23 *Corydalis* species based on *matK* region

**FIGURE S11** The MP tree of 23 *Corydalis* species based on *matK* region

**FIGURE S12** The NJ tree of 23 *Corydalis* species based on ITS2 region

**FIGURE S13** The ML tree of 23 *Corydalis* species based on ITS2 region

**FIGURE S14** The MP tree of 23 *Corydalis* species based on ITS2 region

**FIGURE S15** The NJ tree of 23 *Corydalis* species based on ITS+*matK* regions

**FIGURE S16** The ML tree of 23 *Corydalis* species based on ITS+*matK* regions

**FIGURE S17** The MP tree of 23 *Corydalis* species based on ITS+*matK* regions

**FIGURE S18** The NJ tree of 23 *Corydalis* species based on ITS2+*matK* regions

**FIGURE S19** The ML tree of 23 *Corydalis* species based on ITS2+*matK* regions

**FIGURE S20** The MP tree of 23 *Corydalis* species based on ITS2+*matK* regions
